# Protein Traffic Disorders: an Effective High-Throughput Fluorescence Microscopy Pipeline for Drug Discovery

**DOI:** 10.1038/srep09038

**Published:** 2015-03-12

**Authors:** Hugo M. Botelho, Inna Uliyakina, Nikhil T. Awatade, Maria C. Proença, Christian Tischer, Lalida Sirianant, Karl Kunzelmann, Rainer Pepperkok, Margarida D. Amaral

**Affiliations:** 1 University of Lisboa, Faculty of Sciences, BioISI - Biosystems & Integrative Sciences Institute, Campo Grande, C8, 1749-016 Lisboa, Portugal; 2 Cell Biology and Biophysics Unit, European Molecular Biology Laboratory (EMBL), Meyerhofstraβe 1, 69117 Heidelberg, Germany; 3 Advanced Light Microscopy Facility, European Molecular Biology Laboratory (EMBL), Meyerhofstraße 1, 69117 Heidelberg, Germany; 4 Department of Physiology, University of Regensburg, Universitätsstraße 31, 93053 Regensburg, Germany

## Abstract

Plasma membrane proteins are essential molecules in the cell which mediate interactions with the exterior milieu, thus representing key drug targets for present pharma. Not surprisingly, protein traffic disorders include a large range of diseases sharing the common mechanism of failure in the respective protein to reach the plasma membrane. However, specific therapies for these diseases are remarkably lacking. Herein, we report a robust platform for drug discovery applied to a paradigmatic genetic disorder affecting intracellular trafficking – Cystic Fibrosis. This platform includes *(i)* two original respiratory epithelial cellular models incorporating an inducible double-tagged traffic reporter; *(ii)* a plasma membrane protein traffic assay for high-throughput microscopy screening; and *(iii)* open-source image analysis software to quantify plasma membrane protein traffic. By allowing direct scoring of compounds rescuing the basic traffic defect, this platform enables an effective drug development pipeline, which can be promptly adapted to any traffic disorder-associated protein and leverage therapy development efforts.

It is estimated that more than one fourth of the human genome encodes membrane proteins^1^ which are essential molecules in the cell mediating interactions with the external milieu (channels, receptors, anchors, transporters, sensors, adaptors, etc) and thus targets of over 50% of all modern pharmaceutical drugs. Expectedly thus, mutations leading to defective traffic of membrane proteins and consequent loss-of-function result in a large range of so-called human trafficking disorders [reviewed in Refs. 2, 3]. Frequently, such mutations lead to protein folding defects which trigger degradative mechanisms. So, a large number of protein conformational disorders are also trafficking diseases^4^. Indeed, misfolded plasma membrane (PM) protein conformations are usually recognized by the endoplasmic reticulum (ER) quality control (ERQC) which causes their ER retention and triggers subsequent degradation via the ubiquitin-proteasome pathway (UPP). Examples of such disease mechanism occur widely and include Cystic Fibrosis (CF)^5^, nephrogenic diabetes insipidus^6^, oculocutaneous albinism^7^, early-onset severe obesity^8^, retinitis pigmentosa^9^, Leydig cell hypoplasia^10^, familial hypercholesterolemia^11^ and α_1_-antitrypsin deficiency^12^. In several of these trafficking diseases the mutant protein is residually active but is precluded from exerting its function because it cannot reach the cell surface due to a stringent ERQC.

Pharmacological correction of the basic traffic defect is thus essential to the development of effective therapeutics for these diseases. The specificity of each disorder coupled to the relatively small market size in a rare/orphan disease due to low patient numbers are the main causes for the lack of appropriate experimental strategies and delayed development of therapies for trafficking disorders. Successful drug development for this group of diseases must substantially rely on robust high-throughput (HT) pipelines encompassing traffic assays of high sensitivity which can hardly be achieved in the plate reader [reviewed in Ref. 13]. Traffic efficiency (*i.e.* the fraction of proteins successfully reaching their PM location) determined through HT microscopy is a reliable readout that provides such information.

Among protein conformational/trafficking disorders, CF is a paradigmatic example that has led the way to other rare diseases in many aspects, largely because it is the most common monogenic life-shortening condition in Caucasians. CF is caused by mutations in the gene encoding the CF transmembrane conductance regulator (CFTR)^14^, a glycoprotein anion (HCO_3_^−^/Cl^−^) channel expressed at the apical PM of epithelial cells. About 85% of all CF patients worldwide bear a deletion of phenylalanine 508 (F508del), which leads to CFTR misfolding, ER retention and premature degradation via the ERQC^15^. Additionally to the traffic defect, the residual amount of F508del-CFTR that reaches the PM has very low activity^16^ and fast turnover^17^. The predominant cause of disease morbidity and mortality occurs at the respiratory level, where airway obstruction and cycles of chronic airway inflammation and bacterial infections lead to progressive lung deterioration.

Intensive efforts towards the development of CFTR-targeting therapies have been made in recent years, particularly via HT screening (HTS) projects^18^. These already led to the development of VX-770, the first and only FDA- and EMA-approved CFTR modulator, but only for the G551D-CFTR mutation^19^ and another 8 mutations^20^, which yet altogether only account for ~5% of all CF patients. Notwithstanding, rescuing the most frequent loss-of-function CFTR mutation (F508del) has remained a difficult task. Indeed, the first molecule significantly correcting F508del-CFTR folding *in vitro* undergoing clinical trials – corrector VX-809^21^ – showed only modest effects^22^. One likely cause for this lack of success is the implementation of screening platforms which do not rely on a traffic assay as their primary readout. An attractive alternative would be the case where such a cellular assay would be implemented with a traffic readout amenable to up-scaling for HTS.

Within this background, we report here the establishment of a traffic-based assay on the physiologically relevant CFBE (CF Bronchial Epithelial) cell line stably transduced to express an mCherry-Flag-CFTR traffic reporter for either wild type (wt) or F508del-CFTR under an inducible promoter. This assay is a significant improvement over the previously developed A549 alveolar epithelial cell line^23^ and other alternatives, constituting a reliable platform for drug discovery or identification of therapeutic gene targets. Firstly, because CFBE cells are indeed an excellent preclinical model of CF with high biomedical predictive value, as they are derived from a F508del-homozygous CF patient but with negligible expression of endogenous CFTR^24^. Secondly, the double-tagged reporter allows for ratiometric readouts of traffic efficiency on a single cell basis. Finally, the inducible promoter enables clean assessment of compound (or siRNA) effects on protein biogenesis and early secretory pathway without background interference of any pre-existing protein. Also, this genetic strategy allows quantifying the role of individual genes to CFTR traffic by inducing CFTR expression only after the gene of interest has been knocked-down (*e.g.* via siRNA treatment). Coupling this technology to systematic siRNA knock-downs^25^ or treatment with chemical compound libraries allows pinpointing lead drug compounds/therapeutic genes targets that rescue F508del-CFTR to the PM and/or enhance traffic of wt-CFTR. Our results demonstrate the robustness and sensitivity of the assay in high-throughput mode.

## Results

### CFTR traffic reporter

**Figure 1:**
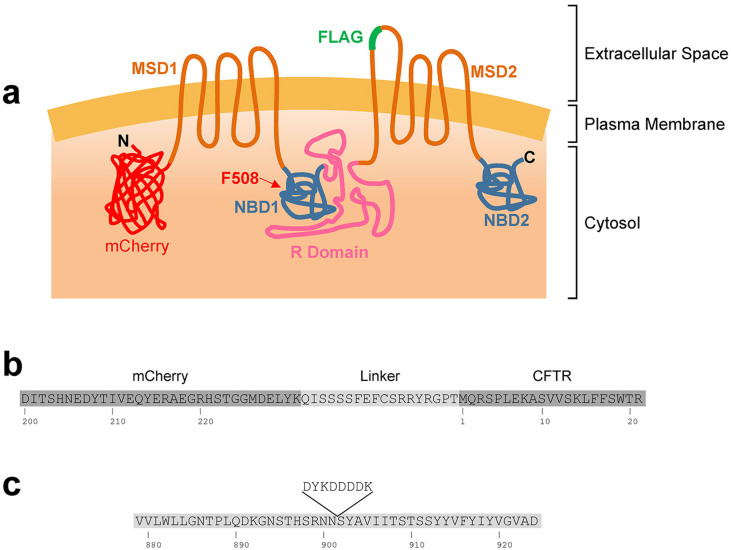
Schematic representation of the CFTR traffic reporter construct. (a) Topology of the CFTR molecule showing the two membrane-spanning domains (MSD), the two nucleotide binding domains (NBD) and the regulatory domain (R Domain). Phe508 locates in the first NBD domain. mCherry was fused to the N-terminus of CFTR via a short linker sequence. The Flag-tag was introduced in the fourth extracellular loop and becomes accessible to the extracellular space when the construct is inserted in the plasma membrane. Both wt and F508del variants were generated. (b) Amino acid sequence showing the linker (light gray shadow) connecting mCherry and CFTR (dark gray shadows). (c) CFTR amino acid sequences showing the insertion of the Flag octapeptide between Asn901 and Ser902.

**Figure 2:**
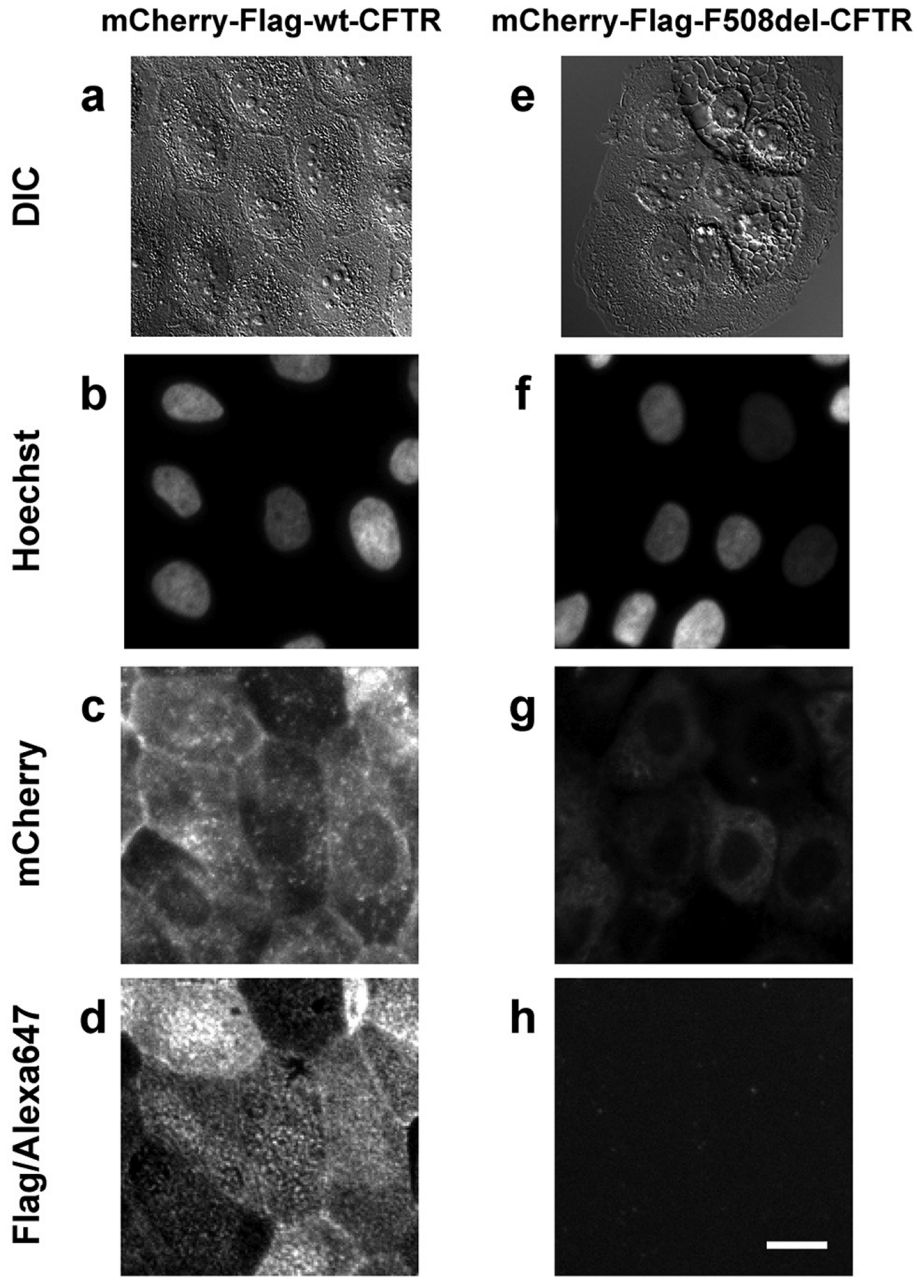
Microscopy features of the CFBE cell lines expressing mCherry-Flag-wt-CFTR (a–d) or mCherry-Flag-F508del-CFTR (e–h). Cells were grown in the presence of 1 μg/ml Dox so as to induce CFTR expression. DIC bright field images (a, e) showing an overall cell morphology reminiscent of CFTR localization, especially for F508del-CFTR, where its ER accumulation is visible. Widefield fluorescence images show triply labelled unpermeabilized cells. Nuclei are stained with hoechst 33342 (b, f), mCherry fluorescence is proportional to the total amount of expressed CFTR (c, g) and Alexa Fluor® 647 (immuno)fluorescence is proportional to the amount of Flag tags exposed extracellularly (*i.e.* CFTR molecules present at the PM) (d, h). Fluorescence images were obtained under equivalent conditions for both cell lines. Scale bar = 20 μm.

The expression of mCherry-Flag-CFTR traffic reporter (Fig. 1) is noticeable after 6 h and quite significant levels are observed after 24 h or 48 h of induction with 1 μg/ml doxycycline (Dox) in wt- (Fig. 2a–d) or F508del-CFTR CFBE expressing cells, respectively (Fig. 2e–h). The Flag-tag only becomes exposed to the extracellular environment and detectable via immunofluorescence – without cell permeabilization – when the construct is inserted in the PM, as it is the case of wt- (Fig. 2d) but not F508del-CFTR expressing cells (Fig. 2h). These data demonstrate that the double-tagged reporter when expressed in CFBE cells reliably recapitulates the widely described trafficking defect associated with F508del-CFTR^5^. The CFTR traffic efficiency can then be determined in individual cells using a ratiometric (Alexa Fluor® 647/mCherry) fluorescence microscopy-based measurement (see below).

### Electrophysiological characterization

**Figure 3:**
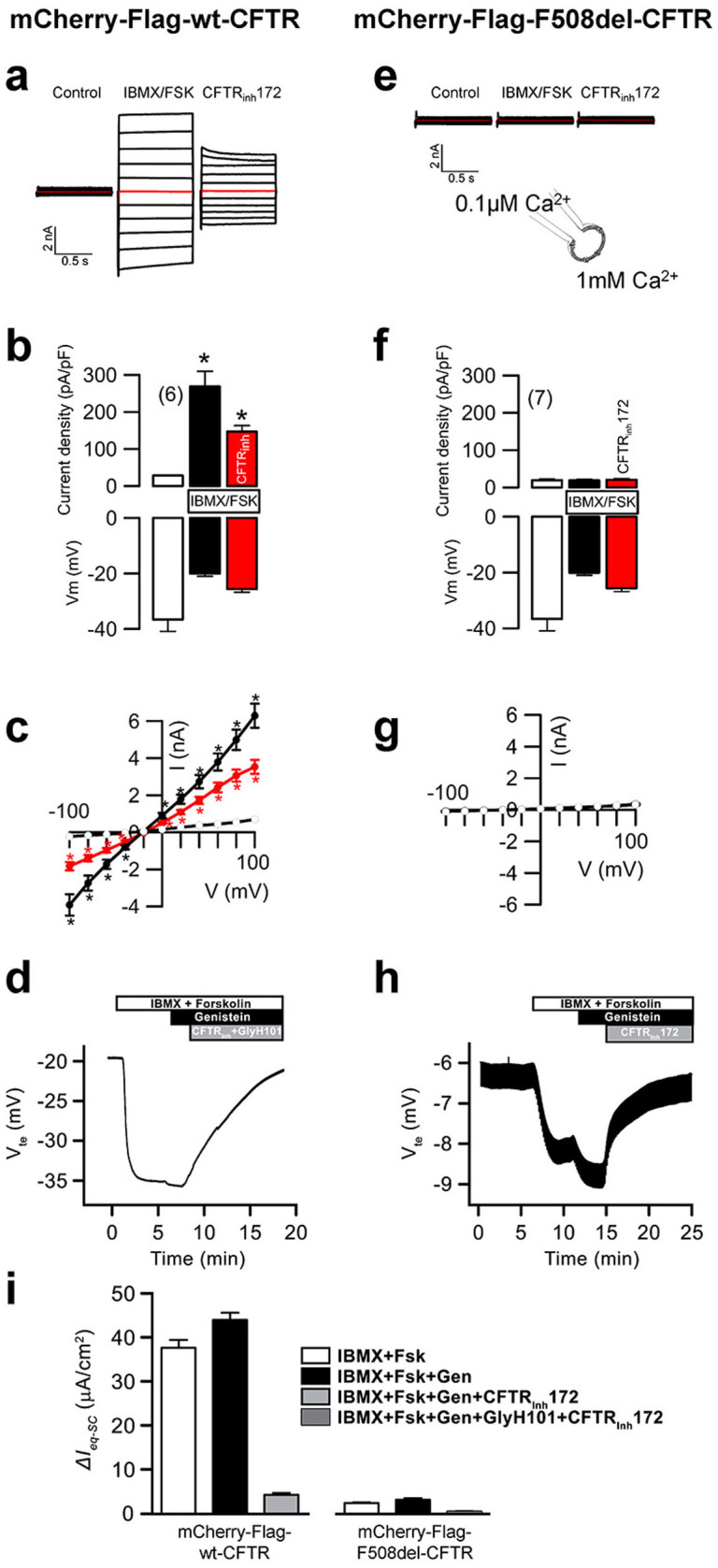
Electrophysiological characterization of the CFBE cell lines. Characterization of the chloride transport activity of mCherry-Flag-wt-CFTR (a–d, i) and mCherry-Flag-F508del-CFTR (e–i). (a) Overlay currents indicating pronounced wt-CFTR whole cell currents after stimulation with IBMX (100 μM) and forskolin (2 μM), and potent inhibition by CFTR_inh_172 (10 μM). Summary of current densities and membrane voltages (b) and current/voltage relationships (c). White circles: unstimulated cells; black circles: cells stimulated with IBMX/forskolin; gray circles: stimulated cells after inhibition with CFTR_inh_172. Mean ± SEM (number of cells). (d) wt-CFTR-dependent transepithelial chloride transport measurement in an Ussing chamber experiment. Chloride secretion was promoted by forskolin and inhibited by a cocktail of CFTR_Inh_172 and GlyH101. (e) Overlay currents indicating lack of current activation of F508del-CFTR. Summary of current densities and membrane voltages (f) and current/voltage relationships (g). Mean ± SEM (number of cells). *significant activation by IBMX/forskolin and inhibition by CFTR_inh_172 (paired t-test). (h) F508del-CFTR-dependent transepithelial chloride transport measurement in an Ussing chamber experiment. (i) Summary of wt- and F508del-CFTR-dependent equivalent short circuit currents as derived from Ussing chamber measurements.

To further validate the physiological relevance of this cellular model as a *bona fide* platform for CF drug discovery pipeline, we next characterized the activity of these double-tagged CFTR constructs for their ability to conduct chloride (Cl^−^). The patch-clamp technique demonstrated that cells expressing mCherry-Flag-wt-CFTR exhibit large whole-cell currents upon stimulation with IBMX (100 μM) and forskolin (Fsk, 2 μM), which were inhibited by CFTR_inh_172 (10 μM) (Fig. 3a–c). Consistently, cAMP-dependent Cl^−^ conductance was also detected in polarized cell monolayers in Ussing chamber experiments (Fig. 3d,i). In contrast, and as expected, no whole-cell currents were detected in F508del-CFTR expressing cells (Fig. 2e–g), similarly to observations in Ussing chamber experiments (Fig. 3h–i). These functional data demonstrate that the double-tagged constructs preserve the CFTR functional integrity and likely its multiple protein interactions.

### siRNA screening platform

**Figure 4:**
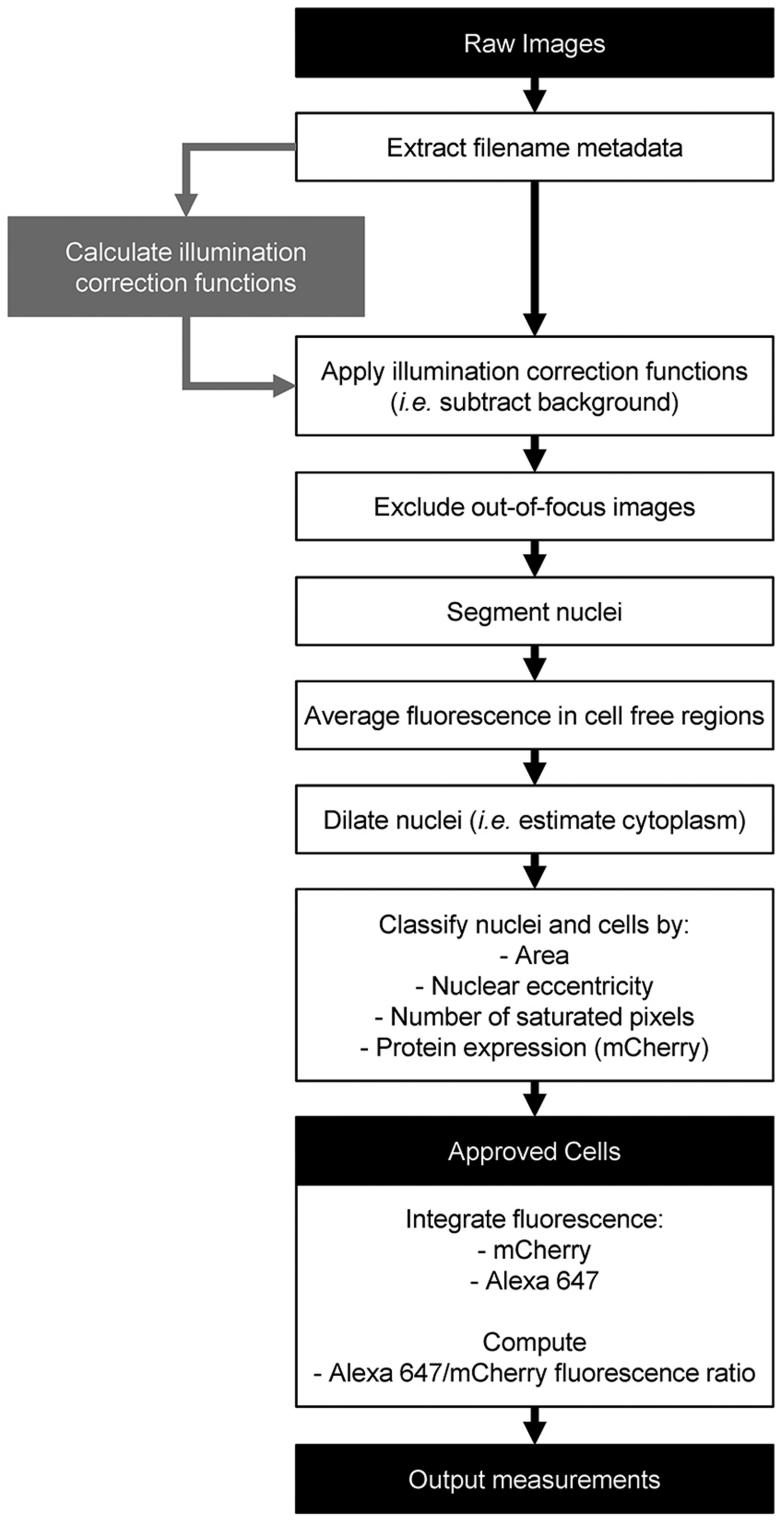
Schematic representation of the image quantification algorithm. Two pipelines were implemented under the CellProfiler software. One of them (gray rectangle) generated the illumination correction functions for the three fluorescence channels (Hoechst, mCherry and Alexa Fluor® 647). The other pipeline (black outlined rectangles) performed background subtraction, cell segmentation, fluorescence integration and several quality control steps which exclude cells with aberrant features – such as apoptotic-like nuclei, saturated pixels and low CFTR expression – from the final output. Filenames describe the treatment (*i.e.* compounds or siRNA) cells were subjected to. Average fluorescence in cell-free areas is used to judge background subtraction accuracy.

To assess the suitability of the screening platform to the identification of putative novel CFTR therapeutic target genes we next performed reverse transfection in siRNA pre-coated microscopy plates. After transfection optimization (data not shown) an siRNA-treatment time of 72 h was selected, a consensus time in other studies^18^,^26^. Expression of the mCherry-Flag-wt-CFTR construct was induced during the last 24 h of siRNA treatment. Image quantification was performed with CellProfiler using two analysis pipelines. One of the pipelines calculated the illumination correction functions (for background subtraction) and a second pipeline performed background subtraction, cell segmentation, fluorescence integration and basic quality control (Fig. 4).

**Figure 5:**
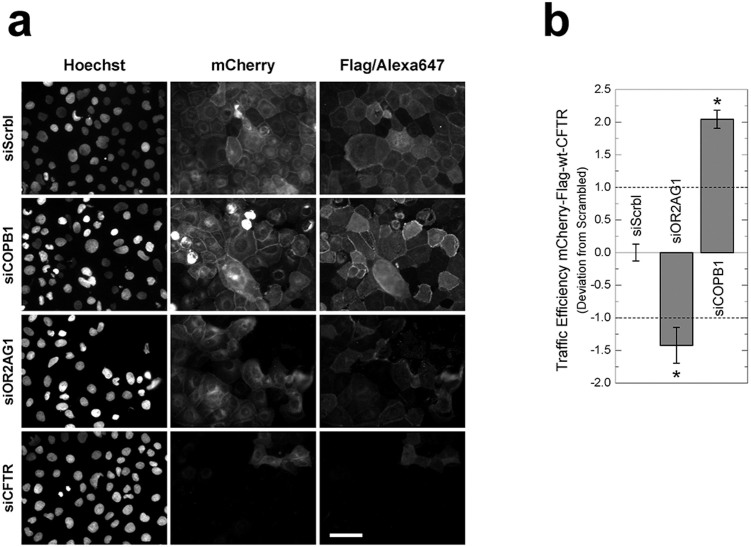
Example widefield epifluorescence microscopy images obtained from the CFTR traffic assay. CFBE cells expressing the mCherry-Flag-wt-CFTR construct were treated with distinct siRNAs (see methods) and Flag-tags stained (a). As a negative control, cells were treated with a non-targeting siRNA (Scrambled/siScrbl). Treatment with a siRNA targeting CFTR itself (siCFTR) highlights the specific detection of CFTR and the high transfection efficiency. Traffic of mCherry-Flag-wt-CFTR was significantly enhanced by knocking down COPB1 (siCOPB1) and significantly decreased by knocking down OR2AG1 (siOR2AG1). Scale bar = 50 μm. Images were quantified to determine traffic efficiency (Formula 1, see methods) (b). Data is presented as the median deviation to negative controls ± SEM (Formula 2). Horizontal dashed lines represent the threshold defined in Formula 2. * p < 0.01 (t-test).

Screening a pilot siRNA library with the mCherry-Flag-wt-CFTR cell line revealed several siRNAs significantly affecting CFTR traffic, as shown in representative immunofluorescence images (Fig. 5a). After expression induction of mCherry-Flag-wt-CFTR, a significant amount of CFTR is detected at the PM in the negative control assay, *i.e.*, “Scrambled” siRNA (siScrbl) treated cells. The sensitivity and dynamic range of this assay are shown by the significant changes in the fluorescence ratio of PM versus total CFTR, which reports on CFTR traffic efficiency of representative siRNAs enhancing or inhibiting wt-CFTR traffic (Fig. 5b). In our pilot screen, we identified COPB1 siRNA as a reproducible wt-CFTR traffic enhancer and the siRNA targeting the OR2AG1 olfactory receptor as a traffic inhibitor. Treatment with CFTR-siRNA (siCFTR) significantly decreased the fluorescence signal in almost all cells, indicating a high transfection efficiency as well as correct association of the Alexa Fluor® 647-fluorescence signal to mCherry-CFTR expression. Although the expression level is not homogenous across all cells, traffic efficiency is robust to such variations due to the ratiometric measurement.

### Compound screening platform

**Figure 6:**
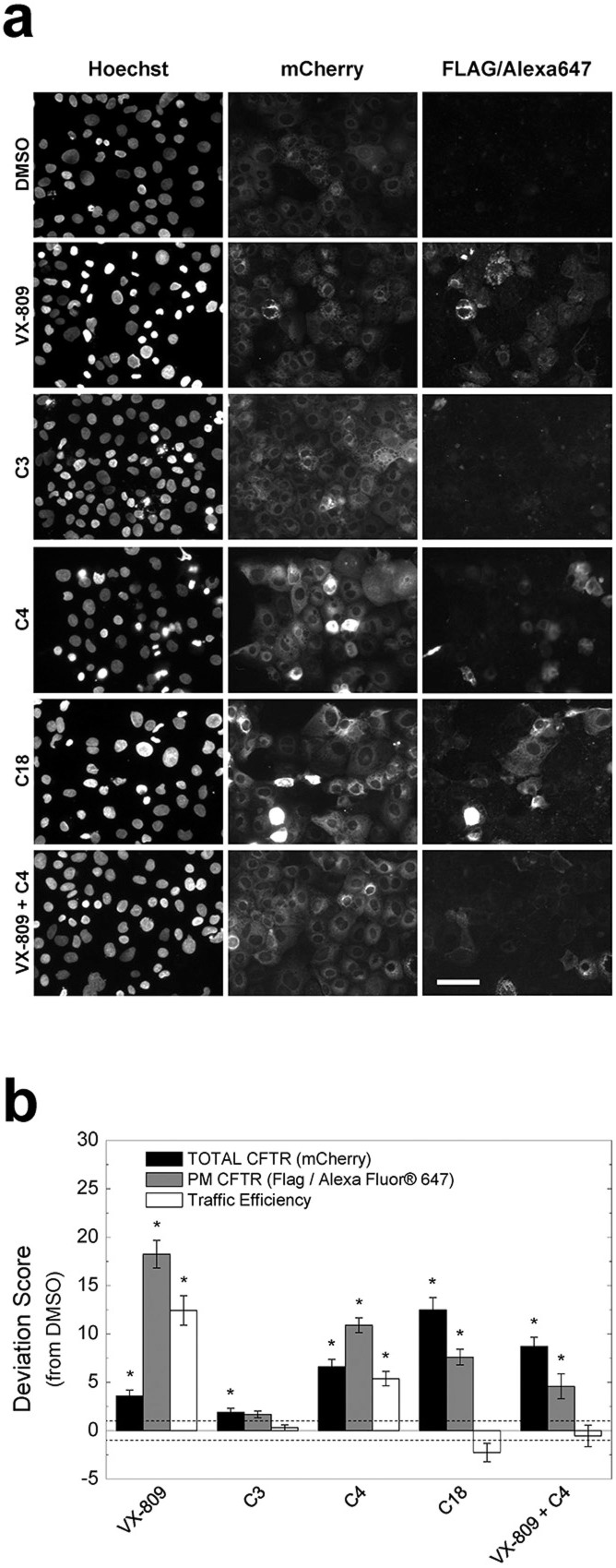
Small molecule screening assay with CFBE cells expressing the mCherry–Flag-F508del-CFTR construct. Cells were treated with DMSO, VX-809 (3 μM), C3 (10 μM), C4 (10 μM), C18 (10 μM) or VX-809 plus C4. Immunofluorescence images of mCherry-CFTR and extracellularly exposed Flag-tags (a) were quantified so as to determine total CFTR expression, PM CFTR amounts and traffic efficiency (total/PM CFTR) versus the DMSO negative control (b). From all tested compounds VX-809, used in isolation, was the most effective rescuing F508del-CFTR traffic. The deviation score is calculated according to Formula 2. Scale bar = 50 μm. Data is presented as median ± SEM. Horizontal dashed lines represent the threshold defined in Formula 2. *p < 0.01 (t-test).

To also determine the sensitivity of the mCherry-Flag-F508del-CFTR cell line to CFTR traffic rescue by small molecules, we performed the traffic assay in the presence of known CFTR correctors: VX-809, C3, C4 and C18 (Fig. 6). Despite some cell-to-cell variation (Fig. 6a), CFTR expression and PM levels were quantified from immunofluorescence images and the corresponding traffic efficiency was calculated (Fig. 6b). Although all compounds significantly increased total CFTR steady-state levels versus DMSO controls – albeit to distinct extents – this did not translate into equivalent PM levels or traffic efficiency increases. Using the traffic assay we were able to detect statistically significant increases in PM F508del-CFTR levels in cells treated with VX-809, C4, C18 or the combination VX-809+C4. As to the traffic efficiency, it was only significantly increased in cells treated with VX-809 or C4 alone. Since VX-809 (3 μM) yielded the highest amount of F508del-CFTR at the PM, this condition appears as the most effective positive control for future screening purposes.

## Discussion

### Overall features of the screening platform

Herein we report the development of a new platform for drug discovery using CF as a model traffic disorder. The currently reported assay is based in the determination of CFTR traffic efficiency (normal protein) or PM levels (mutant protein) and has been designed for high-throughput screening (HTS) conditions. The platform includes cell lines expressing an inducible CFTR (wt or F508del) traffic reporter, a traffic assay adequate for microscopy-based HTS and an automated quantification method. We have validated this platform by performing a small scale siRNA pilot screen and compound-based tests. The siRNA screens showed that the assay specifically detects CFTR and can be used to identify CFTR traffic regulators (potential drug targets). The results with compounds demonstrate the efficient detection of F508del-CFTR at the PM following treatment with the investigational drug VX-809 and C4 (a corrector 4a analogue). In the context of CF, such information allows for: *(i)* identification of regulators of normal and mutant CFTR traffic and potential drug targets; *(ii)* direct discovery of lead compounds rescuing mutant CFTR; *(iii)* development of compounds modulating drug targets of highest therapeutic potential; *(iv)* gaining insight into mechanisms of basic cell biology processes. This platform is currently being used in our laboratory to identify novel CFTR traffic regulators and compounds rescuing F508del-CFTR traffic, as well as traffic regulators of other membrane proteins.

### Comparison to alternative methodologies

Regarding comparison with previously described alternative screening methods for CFTR, these include a fluorogen-activating protein (FAP) based method developed by Holleran *et al* to selectively label a CFTR construct at the PM in living cells and study CFTR endocytosis^27^,^28^ and a variation of this method employs cell-permeant fluorogens to label total CFTR^29^. However, the impossibility to simultaneously labelling both total and PM CFTR hampers a direct measurement of CFTR traffic efficiency and leads to loss of assay sensitivity.

The major advantages of this screening platform versus other alternative methods^27^,^29^,^30^ are 3-fold. Firstly, the conditional (Tet-inducible) expression of CFTR – or other protein of interest – allows assessing the consequences of down- (or up-) regulating the expression of individual genes (via siRNA or cDNA overexpression) or adding compounds before the protein of interest (*e.g.* CFTR) is expressed; this allows detecting the effects of the siRNAs/compounds on the biogenesis of the protein of interest and the early stages of secretory traffic. Secondly, the double-tagged constructs allow for the *simultaneous* readout of the total protein expressed in the cell and the fraction at the PM, which in turn allows to compute traffic efficiency based on ratiometric parameters (in the case of normal protein). The combined inspection of all three measurements allows promptly formulating mechanistic hypothesis on the biological processes targeted by conditions tested. Thirdly, in contrast to the plate reader, the microscopy-based approach described here allows for the simultaneous acquisition of several additional cell parameters. Indeed, it also allows determination of total number of cells, cell shape, cell size, thus all allowing a tight quality control of the data and also to perform statistical analyses based on individual cells. Indeed, this quality control can be easily introduced into the pipeline that analyses the microscopy images through the definition of thresholds, *e.g.*, to discard cells with irregular shape, smaller size (apoptosis), with saturated fluorescence due to overexpression so as to increase the robustness and sensitivity of the assay^18^. These possibilities make indeed this approach superior to previously reported methods that also quantify the rescue of CFTR defects in CF, namely those based on the plate reader. The fact that the constructs recapitulate the cellular location and function of the original CFTR protein constitutes a crucial requirement for the physiological relevance of this platform in CF biomedical research.

Several of the current HTS approaches for CF drug discovery are based on a functional assay, whereby the fluorescence quenching of YFP-transfected cells due to iodide influx is used as a proxy for CFTR function under HT screens^26^,^30^, including compound screens^31^. However, the various limitations of such assay include the lack of an airway cell model – the most reliable preclinical CF model – and the artificial halide conductance being measured (iodide rather than chloride, influx rather than secretion) besides possible interferences e.g., sensitivity to ATP and pH.

### Application to other protein traffic disorders

A key point of the screening platform herein described is its capability of leveraging complementary biomedical research projects – *e.g.* focusing on CFTR-related pathologies as well as those where CFTR is a modifier gene – in which CFTR traffic measurements can provide insights on disease mechanisms and drive therapy development. CFTR-related pathologies encompass the range of disorders caused by CFTR mutations, including the airway hydration imbalance modelled by the CFBE cell line (which is also dependent on the activity of ENaC^32^ and other chloride channels^33^,^34^), pancreatic enzyme insufficiency, CF-related diabetes mellitus and obstructive azoospermia^35^. For such cases, the CFTR traffic construct used here can be expressed in representative cell lines and the same screening strategy readily transferred to the determination of CFTR traffic. CFTR has also been described as a modifier gene in many physiological and pathological conditions, for which CFTR traffic measurements can contribute to establishing the influence of CFTR towards the disease phenotype. Similarly to what has been proposed for CF^36^, research projects in such pathologies can assess the feasibility of using combined administration of multiple drugs – including CFTR modulators – to improve therapeutics. Regarding respiratory conditions, examples include two major diseases namely, chronic obstructive pulmonary disease (COPD), asthma – where patients have higher prevalence of CFTR mutations than healthy controls^37^ – or even sinusitis and allergic bronchopulmonary aspergillosis. Importantly, COPD is characterized by decreased CFTR plasma membrane levels in airway epithelial cells^38^, NF-κB-mediated inflammation in alveolar macrophages and neutrophils is apparently regulated by CFTR^39^, alveolar fluid clearance is regulated by adenosine-mediated CFTR-dependent chloride efflux^40^, and defective CFTR induces aggresome formation and CF lung inflammation through ROS-mediated autophagy inhibition^41^. CFTR thus appears as a master regulator of the respiratory epithelium.

Additionally, the same principles we have adopted to assess CFTR traffic can be readily transferrable to studies of other secretory proteins or other protein traffic diseases. Analogous traffic reporters – whereby the protein of interest is fused with a fluorescent protein plus an extracellular tag – can be generated for any PM protein of interest allowing its traffic to be directly measured. In the case of protein traffic disorders, this strategy allows the ultimate cause of disease, rather than a phenotypic proxy, to be measured thereby increasing the reliability of pre-clinical studies. Other protein traffic diseases potentially benefiting with the development of similar traffic constructs are early-onset severe obesity (caused by mutations in MC4R-melanocortin receptor 4) congenital nephrogenic diabetes insipidus (Aquaporin 2 or Arginine Vasopressin Receptor 2^42^), retinitis pigmentosa (rhodopsin^9^), Leydig cell hypoplasia (luteinizing hormone receptor^10^), familial hypercholesterolemia (LDL receptor^43^) and others^44^.

## Conclusions

The CFTR screening platform reported here is an improvement over current strategies for drug development, namely for CF. The success of this platform – a suitable traffic reporter transfected into an appropriate cell model – encourages the development of similar traffic reporters for proteins involved in other traffic disorders and leverage therapy development efforts.

## Methods

### CFTR Constructs and Cell Line Generation

The CFTR traffic reporter was built as previously described^23^ by fusing mCherry to the N-terminus of wt- or F508del-CFTR via a small linker (QISSSSFEFCSRRYRGPT). Additionally, a Flag sequence (DYKDDDDK) was inserted between Asn901 and Ser902, i.e., in the fourth extracellular loop of CFTR (Fig. 1a–c). CFBE cells (CFBE41o-) were stably transduced with lentivirus encoding the mCherry-Flag-wt- or F508del-CFTR traffic reporters under the control of a Tet-ON promoter (generated by ADV Bioscience LLC, Birmingham, AL, USA).

### Cell Culture

CFBE mCherry-Flag-CFTR cells (wt or F508del variants) were cultured in DMEM high glucose (Gibco #41965) supplemented with 10% (v/v) heat inactivated fetal calf serum (Gibco #10106), 2 mM glutamine (Gibco #25030), 1 mM pyruvate (Gibco #11360), 10 μg/ml blasticidin (Invivogen #ant-bl) and 2 μg/ml puromycin (Invivogen #ant-pr-1) at 37°C and 5% CO_2_. Uncoated 10 cm plastic Petri dishes were used (Nunc™ #150350).

### Assessment of CFTR activity by patch-clamp

Cells grown on cover slips were mounted in a perfused bath on the stage of an inverted microscope (IM35, Zeiss) and kept at 37°C. The bath was perfused continuously with Ringer solution (mM: NaCl 145, KH_2_PO_4_ 0.4, K_2_HPO_4_ 1.6, D-glucose 6, MgCl_2_ 1, Ca-gluconate 1.3, pH 7.4) at about 10 ml/min. Patch-clamp experiments were performed in the fast whole-cell configuration. Patch pipettes had an input resistance of 4–6 MΩ, when filled with an intracellular like solution containing (mM) KCl 30, K-gluconate 95, NaH_2_PO_4_ 1.2, Na_2_HPO_4_ 4.8, EGTA 1, Ca-gluconate 0.758, MgCl_2_ 1.034, D-glucose 5, ATP 3. pH was 7.2, the Ca^2+^ activity was 0.1 μM or 1 mM. The access conductance was measured continuously and was 90–140 nS (EPC 9 amplifier, List Medical Electronics, Darmstadt, Germany). In regular intervals, membrane voltages (Vc) were clamped in steps of 20 mV from −100 to +100 mV from holding potential of −60 mV.

### Ussing Chamber Experiments

For Ussing chamber experiments, inducible mCherry-Flag-CFTR CFBE cells (wt or F508del variants) were seeded at approximately 3 × 10^5^ cells/ml onto Costar Transwell® permeable support of pore size 0.4 μm and 1.12 cm^2^ area. Transepithelial resistance (TER) was routinely measured using a STX chopstick electrode (WPI®). CFTR activity was measured using the open circuit technique when a confluent monolayer was formed, as judged by a TER of 1200–1500 Ω/cm^2^. CFTR was activated with a combination of IBMX (100 μM), forskolin (2 μM) and genistein (25 μM). Specific CFTR inhibition was achieved with CFTR_Inh_172 (30 μM) alone or in combination with GlyH101 (50 μM). Values for the transepithelial voltage (V_te_) were referenced to the lumenal epithelial surface. TER was determined by applying intermittent (1 s) current pulses (0.5 μA). The equivalent short circuit current (I_sc_) was calculated according to Ohm's law (I_sc_ = V_te_/TER), with appropriate correction for fluid resistance.

### Preparation of siRNA coated multi-well plates

Multi-well plates (BD Falcon #353962) were coated with customized siRNAs (Silencer® Select, Ambion) for solid-phase reverse transfection adapted from a previously reported protocol^45^. Briefly, an aqueous 0.2% (w/v) gelatin solution was prepared and filtered with a 0.45 μM pore size filter and a 0.4 M glucose solution was prepared in Opti-MEM. Then, a transfection mix was prepared by mixing 1.662 ml of the sucrose/Opti-MEM solution, 969 μl of Lipofectamine 2000 and 969 μl doubly distilled water. This transfection mix was distributed into a 96-conic well plate (35 μl/well, “Plate A”). In parallel, fibronectin was diluted in the 0.2% gelatin solution to a concentration of 1%. This solution was distributed into another 96-conic well plate (96 μl/well, “Plate B”). Then, 5 μl of a 3 μM siRNA solution and 7 μl of the transfection mix (“Plate A”) were incubated in each well of a low volume 384 well plate (“Plate C”). After a 20-min incubation, 7 μl of the fibronectin solution (“Plate B”) were added. 3 μl of the contents of each well in “Plate C” were diluted fifty fold in a 384 deep well plate using doubly distilled water. Finally, 15 μl of each well were transferred to a 384-well imaging plate, lyophilized and stored in an anhydrous atmosphere before cell seeding. A previously reported “Scrambled” non-targeting siRNA^46^ was used as a negative control.

### CFTR Traffic Assay

CFBE mCherry-Flag-CFTR cells were grown to confluence and split (50%). Twenty-four hours later, cells were trypsinized to antibiotic-free medium and seeded in siRNA coated 384-well plates (50 μl/well, 2 × 10^3^ cells/well) using a Multidrop™ Combi peristaltic dispenser (Thermo Scientific #5840300). CFTR expression was induced 48 h (wt-CFTR) or 24 h (F508del-CFTR) after seeding with antibiotic-free medium supplemented with 1 μg/ml doxycycline (Sigma #9891).

Alternatively, a small-scale drug screen was performed in mCherry-Flag-F508del-CFTR expressing cells grown in siRNA-free 8-well chambered coverslips (Nunc™, #155411) according to the same protocol. At the moment of induction, culture medium was supplemented with 3 μM VX-809 (Selleckchem #S1565), 10 μM C3, 10 μM C4, 10 μM C18, 3 μM VX-809 plus 10 μM C4 (Cystic Fibrosis Foundation Therapeutics, Bethesda, MD; USA) or DMSO controls.

### Immunostaining

72 h after seeding, extracellular Flag-tags were immunostained in non-permeabilized cells. After culture medium removal, cells were washed once in ice cold PBS and incubated 1 h at 4°C with monoclonal anti-Flag antibody (2 μg/ml, Sigma-Aldrich # F1804). Then, cells were washed 3 times with ice cold PBS, incubated 20 min with 3% (w/v) paraformaldehyde (PFA) at 4°C and transferred to room temperature for the remaining staining procedure. Cells were then washed three times with PBS and incubated 1 h with an anti-mouse Alexa Fluor® 647 conjugated secondary antibody (2 μg/ml Molecular Probes #A31571). Cells were then washed 3 times with PBS, incubated with a Hoechst 33342 solution (200 ng/ml, Sigma #B2261) 1 h. Finally, cells were washed three times with PBS, immersed in PBS and incubated overnight before imaging.

All solutions were prepared in Dulbecco's PBS freshly supplemented with 0.7 mM CaCl_2_ and 1.1 mM MgCl_2_. Antibody solutions additionally contained 1% (w/v) bovine serum albumin (BSA, Sigma-Aldrich #A9056). All liquid handling was performed with a manual 96 channel pipette liquidator (Liquidator™ 96, Mettler Toledo #17010335). Solution volumes were 15 μl/well for antibodies, 25 μl/well for PFA and 50 μl/well for Hoechst.

### Image Acquisition

Cell imaging was performed at room temperature with automated widefield epifluorescence microscopes. With a Scan̂R microscope (Olympus Biosystems)^46^ equipped with a metal halide light source (MT20), a 12 bit 1344 × 1024 pixel resolution C8484 CCD camera (Hamamatsu) and a 10× UPlanApo objective (Olympus) with a numerical aperture of 0.4. Exposure times at maximum light brightness for Hoechst, mCherry and Alexa Fluor® 647 were 10–20 ms, 450 ms and 850 ms (wt-CFTR) or 15–30 ms, 700 ms and 1000 ms (F508del-CFTR), respectively. The Hoechst channel was used for contrast-based autofocus. Fluorescence images in Fig. 2 were acquired with a Leica DMI6000 B system equipped with a metal halide light source (EL6000) and a DFC365 FX CCD camera (Leica) with a 1392 × 1040 pixel resolution and 12 bit grayscale representation. A 10× HC PL APO objective (Leica) with a numerical aperture of 0.4 was used.

### Image Analysis

Automatic image analysis was performed with open source software tools (CellProfiler, R), using pipelines tailored to the specific application. Initially, overall transfection efficiency was assessed by observing if cells transfected with siRNAs compromising chromosome segregation exhibited mitotic phenotypes^47^. Failure to observe these phenotypes in more than 75% of images implied the rejection of the corresponding plate from analysis. The algorithm for background subtraction comprised (1) the computation of illumination correction functions for each fluorescence channel, which define the pixel-by-pixel fluorescence baseline for each channel as produced by image illumination and background fluorescence; (2) subtraction of the corresponding illumination correction function from each image. The pipeline includes quality control (QC) steps excluding cells which do not significantly express CFTR, have abnormal morphology (*e.g.* apoptotic cells) or contain a significant amount of saturated pixels. This fluorescence quantification data allowed determining CFTR traffic in each cell according to the following formula:
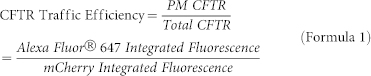


For each image, the CFTR Traffic Efficiency was considered to be the median CFTR Traffic Efficiency for all cells in the image. After imaging at least 4 image fields in triplicate, a custom R script (http://www.r-project.org/) was used to exclude out of focus images and images with high background fluorescence. After averaging the CFTR Traffic Efficiency for all images relating to the same compound or siRNA treatment passing QC (*Traffic Efficiency_Test_*), the effect of different compounds or siRNAs towards CFTR traffic was compared with the one measured under DMSO or “Scrambled” siRNA treatment (*Traffic Efficiency_Neg_control_*) using the following formula:
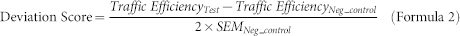
Where *SEM_Neg_control_* is the standard error of the mean for the Traffic Efficiency recorded upon DMSO or “Scrambled” siRNA treatment. We consider significant CFTR Traffic Efficiency effects those whose magnitude is larger than twice the negative control's SEM. Therefore, we define CFTR traffic enhancers as those conditions having a Deviation Score above +1 and CFTR traffic inhibitors as those having a Deviation Score below −1. Additionally, two tailed Student's t-tests were performed to quantify statistical significance versus the corresponding negative control.

## Author Contributions

K.K., R.P. and M.D.A. designed the research. H.M.B., I.U., N.T.A. and L.S. performed experiments. H.M.B., N.T.A., M.C.P., C.T. and L.S. analyzed data. H.M.B., K.K., R.P. and M.D.A. wrote the manuscript.
